# Intranasal Acetaminophen Abuse and Nasal, Pharyngeal, and Laryngotracheal Damage

**DOI:** 10.7759/cureus.5432

**Published:** 2019-08-19

**Authors:** Yufan Lin, Jennifer Y Lu, Carlos D Pinheiro-Neto, David M Jones, Neil Gildener-Leapman

**Affiliations:** 1 Department of Surgery, Division of Otolaryngology, Albany Medical Center, Albany, USA; 2 Department of Pathology, Albany Medical Center, Albany, USA

**Keywords:** acetaminophen, intranasal drug abuse, polarizable talc, septum perforation, subglottic ulceration

## Abstract

A young adult female originally presented with necrosis of the nasal cavity mucosa and septum after sniffing crushed acetaminophen. She underwent endoscopic sinus surgery and debridement but continued to use acetaminophen intranasally. Four months later, the destruction had extended to include the posterior pharyngeal wall and subglottis. The diagnosis was confirmed by polarizable talc found on biopsy of the subglottis. While nasal insufflation of cocaine and hydrocodone-acetaminophen has been well-documented, intranasal abuse of exclusively acetaminophen is not well understood. This case demonstrates the destructive potential of intranasal acetaminophen use and may help physicians recognize unusual signs and symptoms of intranasal drug abuse.

## Introduction

Nasal insufflation has long been associated with abuse of cocaine and more recently, prescription narcotics. The most commonly prescribed form of prescription narcotic abuse is in the form of hydrocodone-acetaminophen [1]. When these medications are abused via nasal insufflation, there is a well-documented history of necrosis of the nasal septum, soft palate, and hard palate. These patients often present with nasal pain, septal perforations, and non-invasive fungal infection [2]. This report describes a case of intranasal abuse of exclusively acetaminophen leading to damage from the nose down to the subglottis. Intranasal abuse of acetaminophen alone is not well-documented and this case may suggest a new trend in drug abuse. It has been seen in only one case study prior despite growing in popularity in the community [3].

## Case presentation

**Figure 1:**
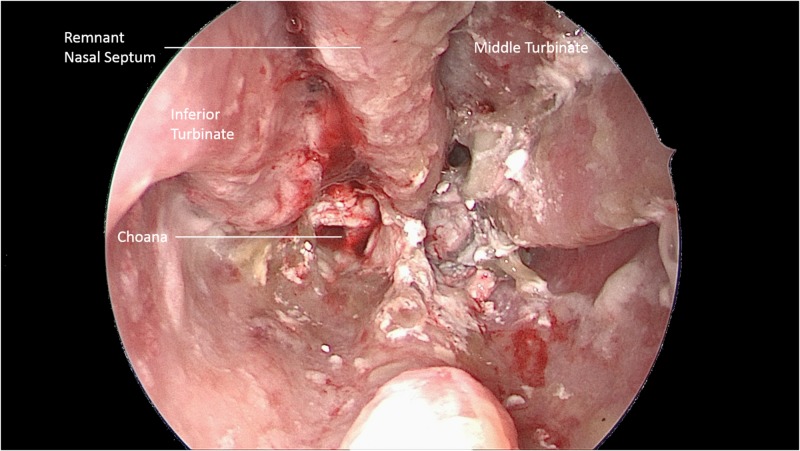
Endoscopic Evidence of Damage Nasal endoscopy obtained with a 0-degree endoscope demonstrating near total septectomy, pill residue, and debris. Diffuse erosive damage was observed along the nasal mucosa with crusted pill debris and blood occluding the choanae and middle meatus.

A young adult female with a history of chronic pain, multi-substance abuse, and obsessive-compulsive disorder originally presented with a history of necrosis of her nasal septum with chronic crusting for greater than six months. She also reported symptoms consistent with Eustachian tube dysfunction, but no hearing loss. At that time, she reported that she was only sniffing crushed over-the-counter acetaminophen. Additionally, she takes a serotonin reuptake inhibitor, but reports she does not snort that. On nasal endoscopy, there was near-complete destruction of the nasal septum with crusting of white powder and secretions in the nasal cavity (Figure 1). A computed tomography (CT) scan of her sinuses demonstrated septal perforation as well as pansinusitis with a mucosal thickening.

**Figure 2:**
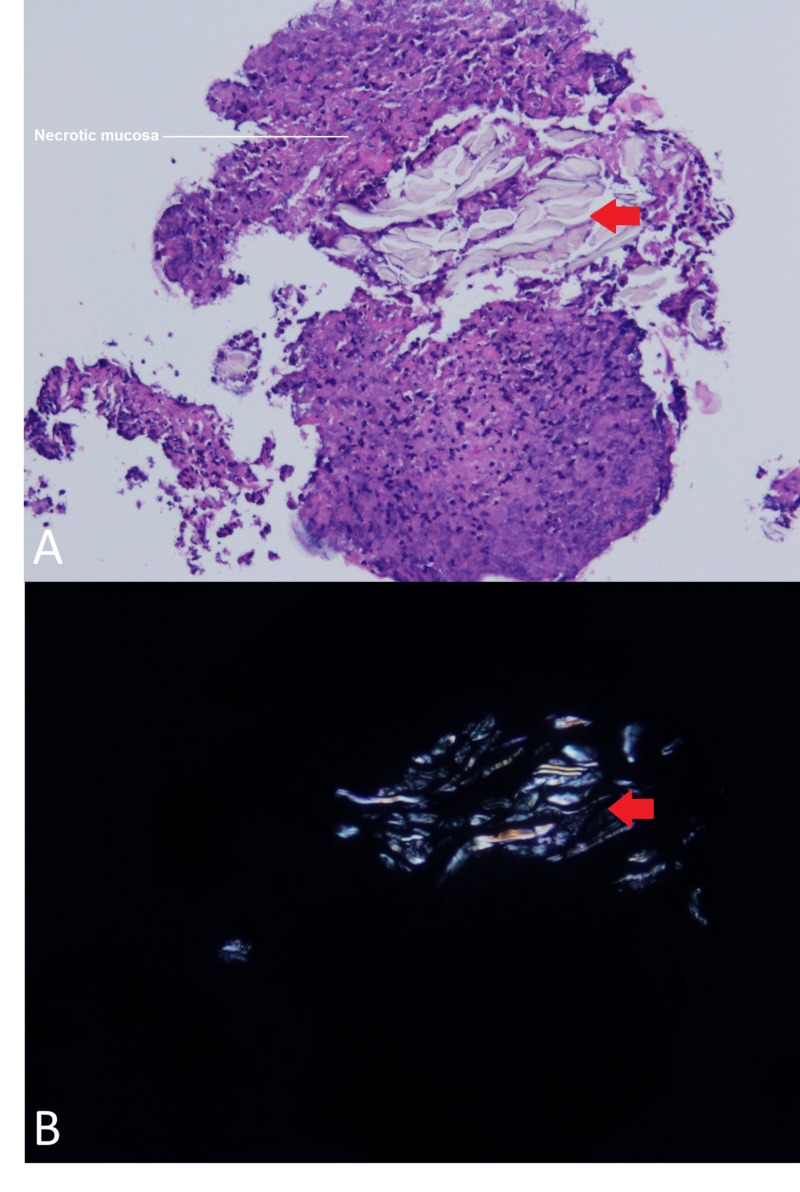
Intranasal Pill Particles on Ulcerated Mucosa on Hematoxylin and Eosin A) 100x magnification of hematoxylin and eosin stained slide showing ulcerated mucosa (white line) and refractile pill material (red arrowhead); B) 100x magnification slide with polarized light highlighting talc fragments from pill remnants.

At this time, the patient was taken to the operating room for endoscopic sinus surgery including bilateral maxillary antrostomy, total ethmoidectomies, and sphenoidotomies. Hematoxylin and eosin stained sections of a nasal mucosal biopsy reveal ulcerated mucosa with attached fibrinopurulent debris and refractile foreign material. Intact portions of mucosa reveal a lichenoid host response and focal subepithelial sclerosis (Figure 2). Her postoperative course was unremarkable, however she continued to use acetaminophen intranasally and was unable to abstain.

She presented to the office four months later with continued postnasal drip, nasal crusting, and eustachian tube dysfunction with a normal audiogram. She also had new complaints of intense pharyngeal pain that interrupted her sleep and periodic hoarseness without dyspnea. She reported a 20 pound weight loss over the past year, with a current body mass index (BMI) of 15. On exam, her voice was hoarse and asthenic. On flexible nasolaryngoscopy, she had recurrent crusting in the nasal cavity and the mucosa was not visible. The nasopharynx and the posterior pharyngeal wall had erosive yellow eschar and there was an anterior subglottic lesion. Important laboratory values include: elevated c-reactive protein at 5.30 mg/L (reference range <3.00 mg/L) and erythrocyte sedimentation rate was elevated at 33 mm/h (reference range 0-20 mm/h). Her white blood cell count was 10.7 x10^9 ^cells/L (reference range 4.0-10.0 x10^9^ cells/L), with elevated absolute neutrophil count at 6.47 x10^9^ cells/L (reference range 1.5-6 x10^9^ cells/L). Her anti-nuclear antibody titer, rheumatoid factor, perinuclear anti-neutrophil cytoplasmic antibody titer, and cytoplasmic anti-neutrophil cytoplasmic antibody titer were all negative.

**Figure 3:**
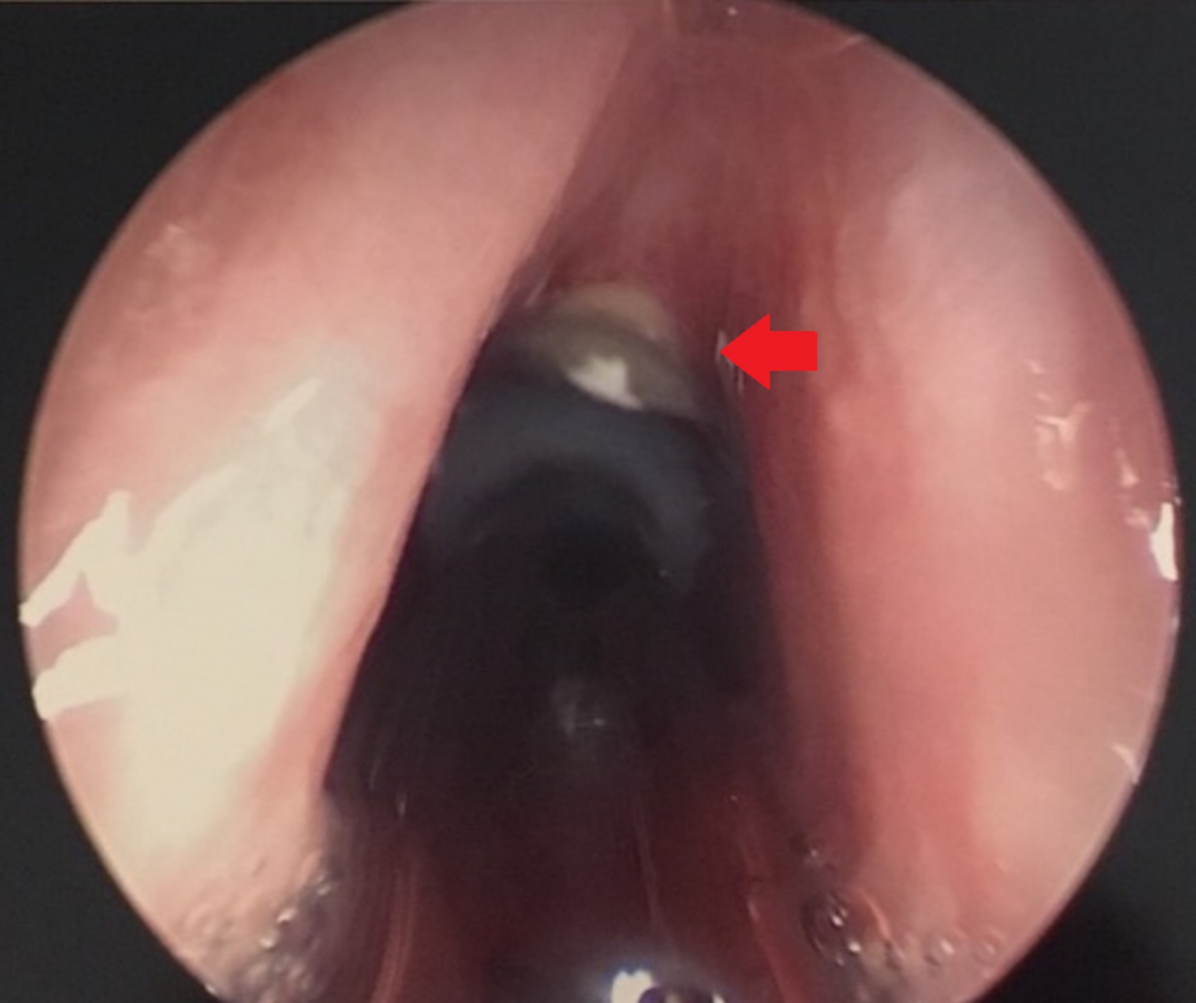
View on Direct Laryngoscopy Anterior subglottic ulcerated tissue and debris (arrowhead).

The patient was educated on nasal humidification, including nasal saline irrigation, as well as intranasal petroleum jelly. In several weeks, despite continued acetaminophen use, her nasal cavity was successfully debrided and normal mucosa was seen throughout the nose. The patient was taken to the operating room for direct laryngoscopy and biopsy (Figure 3). In the operating room, the findings of posterior pharyngeal wall ulceration and subglottic soft tissue lesion were confirmed. Biopsies taken of the posterior pharyngeal wall and subglottis showed a non-ulcerated squamous mucosa with an inflammatory infiltrate and refractile foreign material.

The patient enrolled in a drug abstinence program for several weeks, which allowed for healing of the ulcerated lesions, resolution of pharyngeal pain, and voice complaints. Unfortunately, the patient restarted intranasal acetaminophen use and requires regular in-office nasal debridement, and continues to have some pharyngeal discomfort as well as vocal strain. 

## Discussion

Intranasal abuse of exclusively acetaminophen is not well described in the scientific literature. Studies of intranasal abuse of acetaminophen almost always examine crushed tablets of acetaminophen in combination with another drug, usually hydrocodone. Hardison et al. described a case of exclusively acetaminophen in a 2015 case study of a 34-year-old male, but no other cases can be found [3]. In addition to being a rare documented case of acetaminophen abuse, this case report is unique in the extent of damage, with necrosis and talc expanding into the subglottic region.

The key to recognition of this entity is pathologist and clinician communication about the identification of both non-polarizable and polarizable foreign talc fragments. Talc is used as a tablet binder for acetaminophen and other oral medications; it appears as birefringent, needle-shaped particles under polarized light [4]. Originally, the differential diagnosis for this patient included various disorders causing subglottic inflammation. Autoimmune diseases such as granulomatosis with polyangiitis with sinus and subglottic involvement were considered [5]. Talc on histological finding and negative anti-neutrophil cytoplasmic antibody (ANCA) panel differentiate intranasal drug abuse from the other possible immune etiologies [2].

Acetaminophen is especially appealing because this medication is much easier to obtain, lacking the legal substance controls that regulate cocaine and hydrocodone-acetaminophen. However, whereas the motivation to abuse intranasal opiates is better characterized, abuse of intranasal acetaminophen alone is not well understood. Psychotropic effects including sensations of calm and general feelings of pleasantness have been documented in individual patient experience reports [6]. Pharmacodynamic studies of combined intranasal opiate-acetaminophen use demonstrate faster onset to maximum serum concentrations compared to oral administration as well as slightly elevated peak concentrations [7], which may increase the abuse potential of intranasal use. It is uncertain if our patient derived a high degree of euphoria from her acetaminophen use as she lacked insight about her behavior. We can only speculate about the degree to which her addiction was influenced by perceived benefit versus physiologic effect.

As this method of drug abuse gains popularity, medical professions can expect to see an increase in nasopharyngeal mucosal damage. The physician should also consider potential for hepatotoxicity with acetaminophen used in excess. Long-term follow up of these patients is helpful because abstinence, in this case, was not easy to maintain.

## Conclusions

Even in the absence of prescription and recreational drugs, insufflation should be considered in the differential for nasopharyngeal necrosis. This case report illustrates the intersection of mental health issues and substance abuse involving an easily accessible over-the-counter medication. As this pattern of abuse gains popularity, physicians need to maintain a high index of suspicion for intranasal medication abuse.
